# Methylation and worker reproduction in the bumble-bee (*Bombus terrestris*)

**DOI:** 10.1098/rspb.2013.2502

**Published:** 2014-04-07

**Authors:** Harindra E. Amarasinghe, Crisenthiya I. Clayton, Eamonn B. Mallon

**Affiliations:** Department of Biology, University of Leicester, Leicester, UK

**Keywords:** intragenomic conflict, epigenetics, worker male production, methylation-sensitive AFLP

## Abstract

Insects are at the dawn of an epigenetics era. Numerous social insect species have been found to possess a functioning methylation system, previously not thought to exist in insects. Methylation, an epigenetic tag, may be vital for the sociality and division of labour for which social insects are renowned. In the bumble-bee *Bombus terrestris*, we found methylation differences between the genomes of queenless reproductive workers and queenless non-reproductive workers. In a follow up experiment, queenless workers whose genomes had experimentally altered methylation were more aggressive and more likely to develop ovaries compared with control queenless workers. This shows methylation is important in this highly plastic reproductive division of labour. Methylation is an epigenetic tag for genomic imprinting (GI). It is intriguing that the main theory to explain the evolution of GI predicts that GI should be important in this worker reproduction behaviour.

## 1. Introduction

Genomic imprinting is the inactivation of one allele in diploid individuals, with inactivation being dependent upon the sex of the parent from which it was derived [1]. Imprinting is an evolutionary paradox [2]. Most harmful mutations are recessive. That is, only a single good copy of the gene, out of the two present, is needed for the organism to survive. Why then do organisms sometimes silence one gene when they benefit from a spare? The leading explanation for the evolution of imprinting is Haig's kinship theory. This theory proposes that genomic imprinting arose owing to maternally derived alleles and paternally derived alleles having different selectional pressures with relation to kin resource allocation [1]. Eusocial Hymenoptera are an ideal model system for making independent tests of the theory [1,3–5], as resource allocation in social insect colonies is not just giving resources to offspring, but also has many other components, including sex allocation, caste fate of female larvae, and relevant to our results, male production by workers.

In social insects, reproductive division of labour is not just between the queens and workers, but can actually be between workers. The switch between sterility and reproduction in workers is a much more plastic process than queen–worker differentiation [6]. The kinship theory predicts that there should be conflict between maternally derived alleles and paternally derived alleles of loci involved with worker reproduction. That is, worker reproduction loci should be imprinted [3].

The first step in testing this theory is to search for the molecular mechanism of genomic imprinting at worker reproduction loci. DNA methylation, the addition of a methyl group to a cytosine, is an important genomic imprinting mechanism that is associated with the modulation of gene expression in various eukaryotic organisms [7]. In contrast to the genome-wide methylation found in vertebrates, methylation in insects is sparse and found mainly within genes [8]. Methylation systems are not ubiquitous among insects; CpG methylation is absent in flies (*Drosophila*) [9] and beetles (*Tribolium*) [10]. However, methylation systems appear to be common among the social insects [11]. The honeybee (*Apis mellifera*) was the first insect found to have a fully functioning methylation system [12]. Since then, six ant species have shown evidence of a similar methylation system [13–17]. It has recently been shown that methylation is important in queen–worker differentiation in honeybees [18]. Also in honeybees, the switch between different types of non-reproductive worker roles has been shown to involve DNA methylation [19].

In social insects, there are many potential conflicts, defined as any difference in the reproductive optima of individuals within a society [20]. However, whether these become actual, overt conflicts depends on particulars of the species’ biology [21]. Worker reproduction is rare in honeybees [22]. Worker reproduction in the bumble-bee *Bombus terrestris* is common and makes for a valuable test of methylation's importance in worker reproduction of males. The annual colony life cycle in the bumble-bee is divided into a cooperative phase when the queen has absolute reproductive dominance and a highly aggressive competition phase later in the season when workers and the queen compete over male production [23]. If the queen dies or is removed, workers can be clearly differentiated into reproductive and non-reproductive subcastes by both their ovary development and aggressive behaviour [24].

This paper examines the role of methylation in worker reproduction in *B. terrestris* workers. First, we search for methylation differences between queenless reproductive and queenless and queenright non-reproductive bumble-bee workers using methylation-sensitive amplified fragment length polymorphism (MS-AFLP). This technique detects variation in methylation status of a particular recognition site (5′-CCGG) across a genome [25]. MS-AFLP uses enzymes *Msp*I and *Hpa*II, which have the same recognition site but different sensitivities to cytosine methylation. The methylation state of a particular locus can be detected from the resulting banding pattern. We then ask whether methylation is fundamentally involved in this alteration of reproductive ability. Low doses of 5-aza-2′-deoxycytidine (Decitabine) lead to the passive loss of methylation as the compound is irreversibly bound to DNA methyltransferase [26]. We fed Decitabine to adult and callow worker bees and observed the effects on ovary development and aggression, indicators of the workers taking on a reproductive role [24].

## 2. Material and methods

All experiments were carried out on commercially sourced *B. terrestris* colonies (Koppert Biological Systems, Haverhill, UK). All colonies and bees were kept under red-light conditions at 26°C and 60% humidity on a diet of 50% v/v apiary solution (Meliose–Roquette, France) and fed pollen (Percie du sert, France) ad libitum.

### (a) Methylation differences between different reproductive worker castes

Bumble-bee callow workers (less than 1 day old) from a single colony were reared in three separate boxes, five workers per box. Another four callow workers, captured at the same time, were tagged with a numbered Opalith tag (Christian Graze KG, Germany) and released to the original queenright colony. After 6 days, the bees were sacrificed and the reproduction status of each worker was confirmed by examining the ovaries, as described below (reproducing workers’ oocyte length: mean ± s.d. = 0.6375 ± 0.2459 mm, the bees in the other groups had no discernible ovaries). The bees were immediately stored at −80°C till DNA extractions were performed.

### (b) Methylation-sensitive AFLP

Genomic DNA was extracted from the heads of bees belonging to three different types: a reproductive worker (RW: three bees), a non-reproductive box worker (BW: three bees) and a non-reproductive queenright worker (CW: four bees). The MS-AFLP protocol was modified according to Kronforst *et al*. [11]. Five hundred nanograms of genomic DNA were digested with *Eco*RI and *Msp*I (3 µl of the target DNA, 0.05 µl of *Eco*RI (20 000 units ml^−1^), 0.25 µl of *Msp*I (20 000 units ml^−1^), 1 µl 10× NEB Buffer 4 and 5.7 µl ddH_2_0), and another 500 ng with *Eco*RI and *Hpa*II (3 µl of the target DNA, 0.05 µl of *Eco*RI (20 000 units ml^−1^), 0.5 µl of *Hpa*II (10 000 units ml^−1^), 1 µl 10× NEB Buffer 1 and 5.45 µl ddH_2_0) at 37°C for 3 h.

The products of the two restriction digestions, *Eco*RI–*Msp*I and *Eco*RI–*Hpa*II, were then individually ligated with *Eco*RI adapters (5 µl of EcoRI-F and 5 µl of EcoRI-R at a final concentration of 5 pmol µl^−1^) and *Hpa*II–*Msp*I adapters (25 µl of HpaII-MspI-F and 25 µl of HpaII–MspI-R at a final concentration of 50 pmol µl^−1^), respectively. See the electronic supplementary material, table S1, for the sequences of all adapters and primers. Three microlitres of the digested product were combined with 7 µl of the ligation reaction mixture (1 µl of *Eco*RI adapter (5 pmol), 1 µl of *Hpa*II–*Msp*I adapter (50 pmol), 0.25 µl T4 DNA ligase (400 000 units ml^−1^), 1 µl 10× T4 ligase buffer (New England Biolabs) and 3.75 µl of ddH_2_0) at 37°C for 3 h and then left overnight at room temperature. The ligation products were diluted with 100 µl of ddH_2_0 and used as the template for PCR.

The first PCR (pre-amplification) used 1 µl of ligation product with 1 µl of each EcoRIpre and HpaII-MspIpre primers (10 pmol ml^−1^), and 7 μl of the reaction mix (0.8 μl of 2.5 mM deoxynucleotide triphosphates (dNTPs), 1 μl of 10× Paq5000 Hot Start Reaction Buffer, 0.3 μl of Paq5000 Hot Start DNA Polymerase (500 units), 0.8 μl of 25 mM MgCl_2_, 4.1 μl of sterile distilled H_2_O). The PCR conditions were 94°C for 2 min, followed by 20 cycles of 94°C for 30 s, 60°C for 1 min and 72°C for 1 min, followed by a final extension of 5 min at 72°C.

Seven microlitres of PCR products were then diluted with 93 µl of ddH_2_0 and used as the template for selective amplification. During this step, one of 12 possible selective primer combinations was used. This reduces the number of fragments visualized by gel electrophoresis to a useable number. The selective PCR reaction mixture contained 1 µl pre-amplified product, 1 µl *Hpa*II–*Msp*I primer (10 pmol ml^−1^), 1 µl *Eco*RI primer (10 pmol ml^−1^) and 7 µl reaction mix. Conditions used were 94°C for 2 min followed by 36 cycles (13 cycles of 30 s at 94°C, 30 s at 65°C (0.7°C reduction per cycle thereafter) and 1 min at 72°C followed by 23 cycles of 30 s at 94°C, 30 s at 56°C and 1 min at 72°C) followed by a final extension at 72°C for 5 min before a holding step at 4°C. A schematic of the MS-AFLP is given in the electronic supplementary material, figure S1.

PCR products were diluted with 100 µl of distilled water. Ten microlitres of the diluted amplified product was run on 9% poly(NAT) gels (Elchrom) using the Origins electrophoresis system at 120 V for 81 min at 55°C. The gel was then stained in the dark with SybrGold (1 : 10000 dilution in TAE) followed by a similar destaining step with 100 ml TAE. Bands were scored as either present or absent using Gelanalyzer2010. The resulting matrix was analysed using the R package MSAP [27]. This assesses differentiation between groups by principal coordinates analysis and by analyses of molecular variance (AMOVA).

### (c) Demethylation with 5-aza-2′-deoxycytidine

A stock solution of 5-aza-2′-deoxycytidine (Decitabine) was made by dissolving 5 mg of Decitabine in 2 ml of 1 : 1 v/v acetic acid : distilled water solution. Newly emerged callow workers were collected every day and reared for 7 days in separate Perspex boxes, each box containing a total of five workers. A 10 µM non-lethal dosage of Decitabine (18.5 µl) was added to the apiary syrup (20 ml) of the test group (four boxes, 20 bees in total), whereas the control group (four boxes, 20 bees in total) was fed with unadulterated apiary syrup. Both apiary syrups were coloured using a natural food colourant (green) and four bees taken randomly from four different boxes (two treated and two controlled) were dissected after 2 days in order to confirm that the bees were drinking the solution. New bees were added to these four boxes to make the final number of workers in each box up to five. Fresh solutions (apiary solution and chemical) were provided every day through the experiment. This experiment was repeated for adult workers (more than 1 day old). Unlike the callow bees, these bees were left for three weeks. This was how long it took for clear signs of egg-laying to appear in these adult bees. Behavioural and methylation analyses were only carried out on callow workers, whereas ovary measurements were made on both adults and callows.

### (d) Behavioural effects of demethylation

Three distinct behaviours were recorded in the callow bees as follows. (i) ‘Attack’ included occurrence of one of the following behaviours: biting, pushing, head butting, dragging, wing pulling, struggling or an attempt to sting. (ii) ‘Darting’: a sudden movement of a bee towards another bee but without any body contact between the two bees. (iii) ‘Humming/buzzing’: a series of rapid, short wing muscle vibrations that produce a distinctive buzzing sound. Each group of callow bees was scanned for 10 min three times a day for 6 days at fixed hours (9.00, 13.00 and 17.00) and the frequency of occurrence of each behaviour was recorded. An index of aggression was constructed as the unweighted sum of ‘Attack’, ‘Darting’ and ‘Humming’ that were observed during all the observations throughout the experiment

### (e) Demethylation effects on ovarian development

On days 7 and 21, respectively, callow and adult bees were sacrificed and dissected by making two lateral incisions in the abdomen to observe their ovary development. The ovaries were removed and the length of the largest oocyte in each of the two ovaries was measured as an index of ovary development. All measurements were obtained to the nearest 0.05 mm with an eyepiece micrometer under a dissecting microscope. The length of the largest oocyte in bumble-bees is tightly correlated with a worker's reproductive status [24].

### (f) Amplification of intermethylated sites

Amplification of intermethylated sites (AIMS) is a technique to examine methylation patterns similar to AFLP, but rather than doing the methylation-sensitive/methylation-insensitive digestion in parallel, it does them in sequence. DNA was extracted from each of the 40 callows used. Preparation of adapters was conducted according to [28], 25 μl Blue (100 μM) (5′-ATTCGCAAAGCTCTGA-3′) and 25 μl MCF (100 μM) (5′-CCGGTCAGAGCTTTGCGAAT-3′) were incubated at 65°C for 2 min followed by room temperature for 1 h. The DNA was digested first with the methylation-sensitive restriction endonuclease *Sma*I, which cleaves leaving blunt ends CCC/GGG. One microgram of DNA was digested with 1.5 μl of 10× NEB4 buffer, 0.1 μl *Sma*I (20 000 units ml^−1^) and 3.4 μl ddH_2_O and incubated for 1 h at 25°C. This was then digested with a methylation-insensitive restriction enzyme, *Xma*I (0.5 μl, 10 000 units ml^−1^) for another hour at 37°C with 1.0 μl of 10× NEB4 buffer, 0.5 μl of bovine serum albumin and 8 μl of ddH_2_O [29]. Twenty-five microlitres of this product was ligated to 20 μl of adapter (2 nmol) using 8 μl of 10× T4 buffer and 2 μl of T4 DNA ligase (400 000 units ml^−1^), incubated at room temperature for 10 min. The enzymes were inactivated by incubating the samples for 10 min at 65°C.

The amplification of sequences with the adapters of digested DNA was conducted using the primer sets A (A1, A2), B (B1, A2) and C (C1, C2) (A1, Blue-CCGGGCTA; A2, Blue-CCGGG-TGG; B1, Blue-CCGGGCTG; C1, Blue-CCGGGCGCG; C2, Blue-CCGGGCAAC). Reactions were composed of 12.5 μl YB *Taq* 2× reaction buffer (York BioSciences), 1 μl of each primer (10 pmol ml^−1^), 3 μl of DNA, 0.5 μl of 10 mM MgCl_2_ and 7 μl of water. PCRs with primer sets A and B were composed of 30 two-step cycles: 15 s at 94°C and 60 s at 74°C. The PCR programme for primer set C consisted of 30 three-step cycles: 15 s at 94°C, 45 s at 68°C and 1 min at 72°C. All PCR cycles were preceded by a denaturing step of 95°C for 1 min and ended with an extension of 72°C for 5 min. Ten microlitres of PCR product were run on 9% poly(NAT) gels (Elchrom) using the Origins electrophoresis system at 120 V for 81 min at 55°C. The gel was then stained with SybrGold (1 : 10 000 dilution in TAE). Bands were scored as either present or absent using Gelanalyzer2010. The resulting matrix was analysed using the R package MSAP [27].

## 3. Results

### (a) Methylation differences between different worker reproductive castes

**Figure 1.**
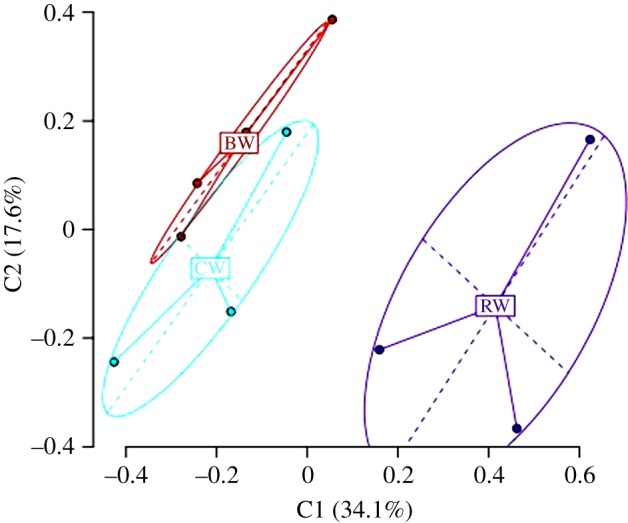
Principal coordinates analysis based on methylation status of loci. RW, reproductive workers; BW, non-reproductive queenless workers; CW, non-reproductive queenright workers. (Online version in colour.)

A total of 245 unique bands (loci) were present. One hundred and thirty-six of these were methylation-sensitive, that is, they showed differences between the digests of *Hpa*II and *Msp*I. Thirty-eight of these were polymorphic, that is, they showed different banding patterns between individuals. There was a significant difference between the methylation status of different worker reproductive castes (*φ*_ST_ = 0.2807, *p* = 0.0045). There was a significant difference between the methylation status of reproducing workers (RW) versus non-reproducing box workers (BW) (*φ*_ST_ = 0.3641, *p* = 0.0259). There were no significant differences between queenless reproducing workers (RW) and queenright non-reproducing workers (CW) (*φ*_ST_ = 0.3572, *p* = 0.0963) nor between BW and CW workers (*φ*_ST_ = 0.06859, *p* = 0.2012). Table 1 details the methylation levels of the three groups. The PCA based on pairwise difference showed three groupings corresponding to their reproductive state (figure 1). The first two axes explain a total of 51.7% of the variation.

**Table 1.** Proportion of each banding type found in each group. HPA−/MSP− was counted as uninformative in MSAP. This is the more conservative approach.

banding pattern	methylation status	BW	CW	RW
HPA+/MSP+	unmethylated	0.35478	0.40931	0.24755
HPA+/MSP−	hemi-methylation of external cytosine	0.05515	0.08088	0.07108
HPA−/MSP+	full methylation at internal cytosine	0.09191	0.10784	0.15686
HPA−/MSP−	full methylation or absence of target	0.49816	0.40196	0.52451

### (b) Effects of 5-aza-2′-deoxycytidine

**Figure 2.**
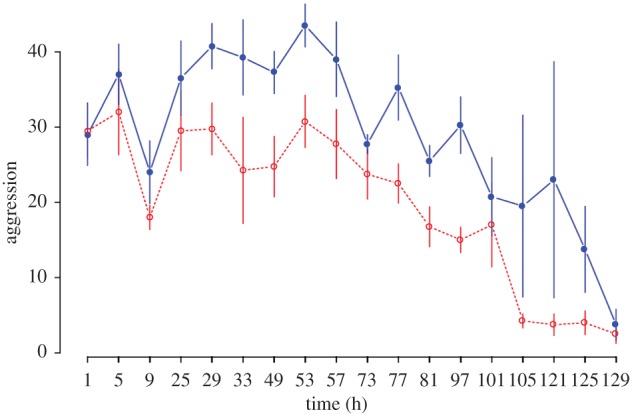
Changes in aggression over time between Decitabine and control callow bees. The filled circles (solid line) represent means of boxes treated with Decitabine. Open circles (dotted line) are the control boxes. Bars represent standard errors. (Online version in colour.)

Aggression is a measure of the reproductive conflict among workers [24]. The dominant hierarchy of bees is usually established through their overt agonistic behaviours. Aggression was analysed using a two-way repeated measures ANOVA, where time was the repeated measure. Decitabine had a significant effect on level of aggression (*F*_1,143_ = 32.17, *p* < 0.00001). Time also had a significant effect (*F*_17,143_ = 8.05, *p* < 0.00001). There was no interaction effect between time and Decitabine (*F*_17,143_ = 0.59, *p* = 0.8957; figure 2).

**Figure 3.**
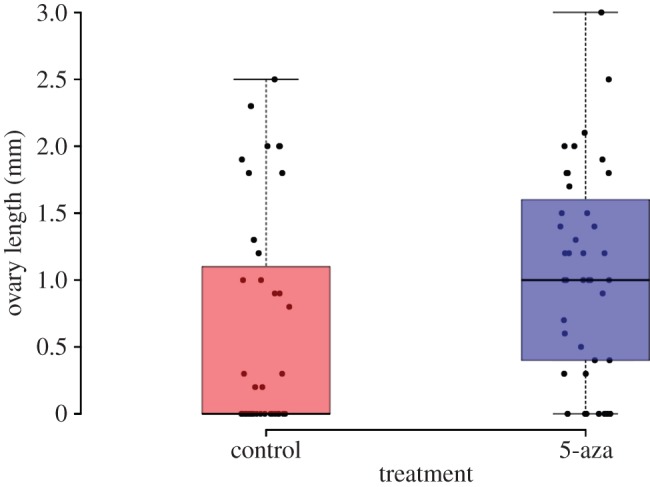
Ovary development between treatments for callow bees. Dots are the individual data points. (Online version in colour.)

Decitabine had different effects on bees’ ovary development depending on whether they were callows or adults when placed in the box (lifestage : treatment: *F*_1,153_ = 7.485, *p* = 0.006957). Decitabine had no effect on level of ovary development in adult bees (*F*_1,75_ = 1.547, *p* = 0.217). Decitabine had a significant effect on level of ovary development in callow bees (*F*_1,75_ = 7.211, *p* = 0.00891; figure 3). On average for the callow workers, each control box had one bee out of five with developed ovaries (more than or equal to 1 mm) [23] compared with three bees out of five with developed ovaries in Decitabine groups.

**Figure 4.**
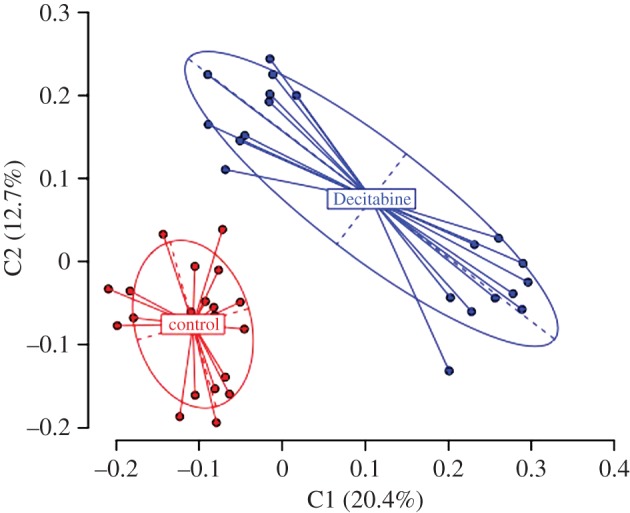
Principal coordinates analysis of AIMS data for callow bees. (Online version in colour.)

In callow bees, Decitabine has a significant effect on methylation patterns based on AIMS data (*φ*_ST_ = 0.2227, *p* < 0.0001). There were 62 loci in total of which 54 were polymorphic. Forty-three loci had the same modal level of methylation in both the control and Decitabine-treated callow workers. Nine loci showed hypermethylation and 10 hypomethylation in Decitabine-treated callows compared with controls. The PCA based on pairwise difference showed two groupings corresponding to whether the bees were treated with Decitabine or not (figure 4).

## 4. Discussion

We found that there were clear differences between the methylation of the genomes of queenless reproductive workers and queenless non-reproductive workers (figure 1). Queenless workers whose genomes had experimentally altered methylation were more aggressive (figure 2) and more likely to develop ovaries compared with control queenless workers (figure 3).

Decitabine had no effect on bees that were adults at the beginning of the experiment. Only callows (bees less than 1 day old) were affected. Although it is tempting to suppose that these adult bees had become developmentally fixed and were unable to switch roles [30], it is just as likely to be a technical artefact of the Decitabine demethylation process and adult insect cell division. Methylation is passively lost in one of the daughter DNA molecules because DNA (cytosine-5)-methyltransferase 1 (Dnmt1) is unavailable to remethylate hemi-methylated sites created during the first round of DNA replication. This occurs because Dnmt1 is inactivated due to covalent linkage to 5-aza-2′-deoxycytidine residues in CpG sites in DNA [26]. Adult insects are considered post-mitotic [31], although see [32]. If no cell division occurs in adult bees, then 5-aza-2′-deoxycytidine cannot be incorporated and there will be no effect on methylation in adult bees. Also it is unwise to compare the callow and adult dataset, as the age, behaviour and methylation status of the adults are unknown and the adult experiment ran for three weeks compared with 7 days for the callow experiment.

The effects of Decitabine are not due to a general toxicity effect. A more general toxic effect would be expected to act on adult bees as well. The adult experiment showed no effects of our dosage of Decitabine. Our dosage is below the minimum used to test for genotoxic effects in *Drosophila* [33]*.* If it were merely a toxic effect, we would expect to see reduced activity and reproduction. This was exactly the result when the pesticide chlorantraniliprole was administered in a similar experimental set-up to ours [34]. Instead, we find increased aggression and more bees becoming reproductive when administered Decitabine.

Decitabine clearly has an effect on methylation patterns in callow workers (figure 4). But it is not obvious that it is a reduction in overall methylation. This could be owing to the limitation of AIMS as a quantifier of exact methylation levels. As there is a PCR step, even if methylation was reduced, rather than completely removed, in a given locus, this locus would be classed as still methylated. Also, it has recently been shown that Decitabine is not a global demethylator but rather demethylates specific and reproducible sites in human cancer cell lines [35]. Nonetheless, Decitabine clearly affects methylation and this affects worker reproduction.

Previous work in honeybees has shown that methylation changes are involved in the switch between workers and queens [18]. The development of a genetically identical embryo into either a queen or a worker has been compared to the transition from a totipotent stem cell to a fully differentiated cell type [30,36]. Recently, it has been shown that bumble-bee workers can reverse their reproductive status depending on the social context [6]. If a reproductive worker is returned to her natal colony, she will regress back to sterility. This is not the case if she is placed in a foreign nest. We have shown worker reproduction to be under epigenetic control. These two observations suggest that worker reproduction is influenced by more plastic epigenetic processes than those of queen–worker differentiation—processes, it has been suggested, that could be analogous to somatic cell reprogramming and transdifferentiation [36].

Haig's kinship theory for the evolution of genomic imprinting predicts that genes involved in queenless worker reproduction should be imprinted [3]. In this paper, as the first step to testing this theory, we have shown that methylation, an epigenetic tag of imprinting in mammals and flowering plants, is important in exactly this behaviour. Our future work will examine parent of origin-dependent monoallelic expression of worker reproduction loci in this highly tractable system.

## Supplementary Material

Table S1 and Figure S1

## Supplementary Material

Raw data
