# Supplementary material for: Methylation and worker reproduction in the bumble-bee (Bombus terrestris)
Source: Proc Biol Sci. 2014 Apr 7;281(1780):20132502. doi: 10.1098/rspb.2013.2502 (PMC4027386; doi:10.1098/rspb.2013.2502)
Supplement: Table S1 and Figure S1 [file rspb20132502supp1.docx]

Supplementary material for **Methylation and worker reproduction in the bumblebee (Bombus terrestris)**

Table S1 : Sequences of ligation adapters, pre and selective amplification primers

| Adapter / Primer | Sequence (5’ -3’) |
| --- | --- |
| Ligation |  |
| *EcoR*I-F | CTCGTAGACTGCGTACC |
| *EcoR*I-R | AATTGGTACGCAGTCTAC |
| HpaII-MspI–F | GACGATGAGTCTAGAA |
| HpaII-MspI –R | CGTTCTAGACTCATC |
| Preamplification |  |
| EcoRIpre (EcoRI + 0) | GACTGCGTACCAATTC |
| HpaII-MspI pre (HpaII-MspI + A) | GATGAGTCTAGAACGGA |
| **Selective amplification** |  |
| **Eco-AA** | **GACTGCGTACCAATTCAA** |
| **Eco-AT** | **GACTGCGTACCAATTCAT** |
| **Eco-AG** | **GACTGCGTACCAATTCAG** |
| **Eco-AC** | **GACTGCGTACCAATTCAC** |
| **HpaII-MspI-AAT** | **GATGAGTCTAGAACGGAAT** |
| **HpaII-MspI-ACT** | **GATGAGTCTAGAACGGACT** |
| **HpaII-MspI-ATC** | **GATGAGTCTAGAACGGATT** |

**
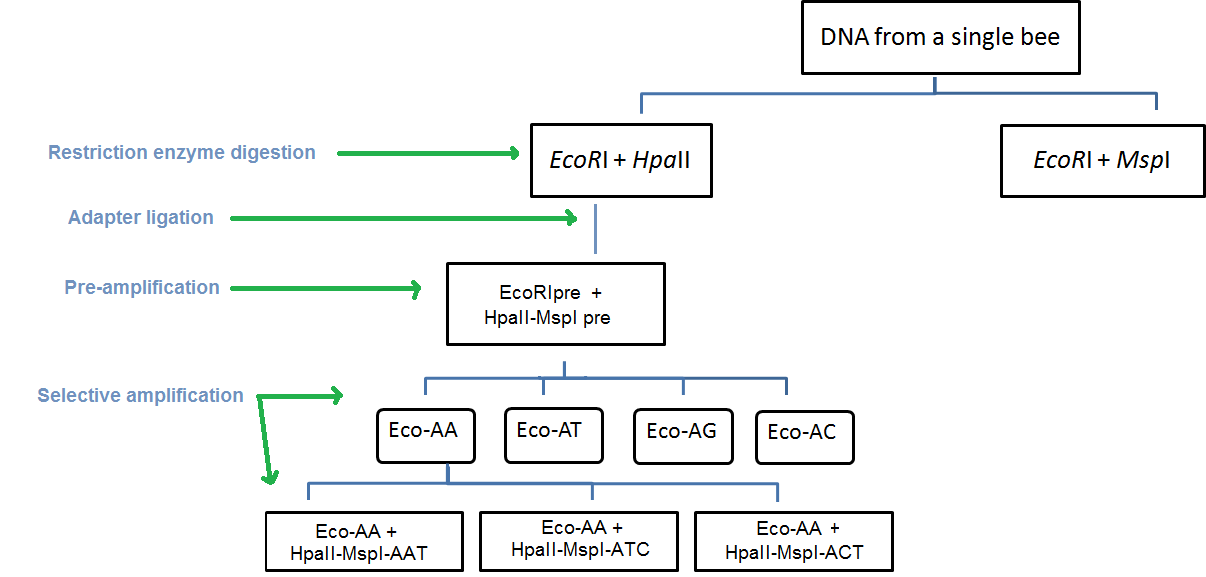
**

**Figure S1: A flow diagram to show different primer and adapter and enzyme combinations used during the MS-AFLP protocol.**
